# Identification and characterization of invasive multi-drug-resistant (MDR) *Bacteroides* genomospecies in Canada

**DOI:** 10.1099/acmi.0.000111

**Published:** 2020-02-26

**Authors:** Christopher Graham, Alireza Eshaghi, Alicia Sarabia, Sandra Zittermann, Patrick Stapleton, Julianne V. Kus, Samir N. Patel

**Affiliations:** ^1^​ Trillium Health Partners, Mississauga ON, Canada; ^2^​ Department of Medicine, University of Toronto, Toronto ON, Canada; ^3^​ Public Health Ontario Laboratory, Public Health Ontario, Toronto ON, Canada; ^4^​ Department of Laboratory Medicine and Pathobiology, University of Toronto, Toronto ON, Canada

**Keywords:** *Bacteroides*, multi-drug resistance, genomospecies, metronidazole, anaerobic bacteria

## Abstract

We identified and characterized a genome of the multi-drug-resistant *Bacteroides* genomospecies recovered from an invasive specimen from a hospitalized patient in Canada. The strain was resistant to penicillin, pipercillin-tazobactam, meropenem, clindaymycin and metronidazole. The strain harboured a plasmid containing the *nimE* gene, which has been shown to be associated with metronidazole resistance. The study highlights the importance of being vigilant in suspecting antimicrobial drug resistance when a patient is not improving on therapy.

## Background

*Bacteroides* species are one of the most abundant and important parts of the normal gut flora in humans. They are also the most significant opportunistic anaerobic pathogen. *Bacteroides fragilis* group, which contains multiple closely related *Bacteroides* species, is the most commonly encountered anaerobe associated with infections. Infections caused by the *B. fragilis* group include intra-abdominal infection, deep-tissue infection and sepsis. These infections can lead to severe outcomes including death if they are not treated appropriately and promptly. Resistance to several antibiotics including penicillin, clindamycin, cefoxitin and moxifloxacin has been well established but resistance to metronidazole, piperacillin-tazobactam and carbapenems remains relatively rare in North America [1, 2].

## Case presentation

A patient with no travel or hospitalization history in the last 5 years, a history of alcohol abuse, recurrent pancreatitis and a complicated ICU admission for pneumonia 7 years prior, was admitted to hospital with pneumonia. The patient was started on azithromycin and ceftriaxone. On day 3, the patient deteriorated, was moved to the ICU and treatment changed to piperacillin-tazobactam. Initially, the patient improved but on day 12, developed diarrhea; metronidazole (for possible *Clostridioides difficile* infection) and meropenem were added. A CT scan revealed evidence of ischaemic bowel with perforation and abscess; the patient underwent a laparotomy for source control. Meropenem was continued for 7 days but metronidazole was stopped after 2 days. During the laparotomy, an intra-abdominal swab was taken, which grew *Bacteroides fragilis *, herein this isolate is referred to as PHL2737. The patient remained afebrile and completed 7 days of meropenem treatment. Two days later, the patient became febrile again. A blood culture from a 17-day-old central line grew *B. fragilis* (peripheral culture was negative) and meropenem was restarted. The patient had ongoing fever and inotrope dependence. The central line was removed and imaging revealed an intra-abdominal fluid collection. A percutaneous drain was placed to channel purulent material, which was sent for culture and grew *B. fragilis *. The patient remained febrile and due to the concern of possible carbapenem resistance, linezolid was added to the meropenem without any additional interventions. The patient subsequently defervesced, inotropes were discontinued and was transferred out of the ICU on day 39 with subsequent discharge home on day 70. Bacterial isolates were sent to Public Health Ontario (PHO) Laboratory for susceptibility testing and confirmation of identification.

Isolate PHL2737 was identified as *B. fragilis* using Bruker MALDI Biotyper and 16S rRNA gene sequencing at PHO Laboratory. Susceptibility results showed extensive resistance to penicillin (MIC≥64 mg l^−1^), meropenem (MIC≥64 mg l^−1^), pipercillin-tazobactam (MIC≥512/4 mg l^−1^), clindamycin (MIC≥512 mg l^−1^) and metronidazole (MIC≥512 mg l^−1^) (CLSI M100-S28). The unusual susceptibility pattern prompted additional investigation.

Genomic DNA (gDNA) was used to prepare an Illumina library using Nextera XT (Illumina, San Diego, CA, USA). The library was sequenced on the MiSeq platform. Oxford Nanopore (Cambridge, MA, USA) libraries were also prepared following the protocol for 1D gDNA long reads using a 1D ligation sequencing kit without fragmentation and including the DNA repair step with the NEBNext FFPE DNA repair module (New England Biolabs, Ipswich, MA, USA). The library was sequenced on the MinION.

A total of 5 473 415 paired-end illumina reads were assembled using *de-novo* assembler in CLC Genomics Workbench version 8.0.1 (Qiagen, Canada); 111 contigs (range from 538 to 384 650 bp; N50=141 340 bp) with an average coverage of 229.5-fold were generated. Using illumina and Nanopore sequences, hybrid *de novo* assembly was performed using Unicycler v0.4.8-beta to close the genome and plasmids. Annotation by NCBI Prokaryotic Genome Annotation Pipeline (PGAP) predicted 4780 CDS, including 90 RNA genes: 6 5S rRNA, 6 16S rRNA, 6 23S rRNA and 72 tRNA genes. The chromosomal sequence of the PHL2737 strain was 5 522 430 bp, which has been deposited into GenBank (GenBank accession no. QAWD00000000).

**Fig. 1.**
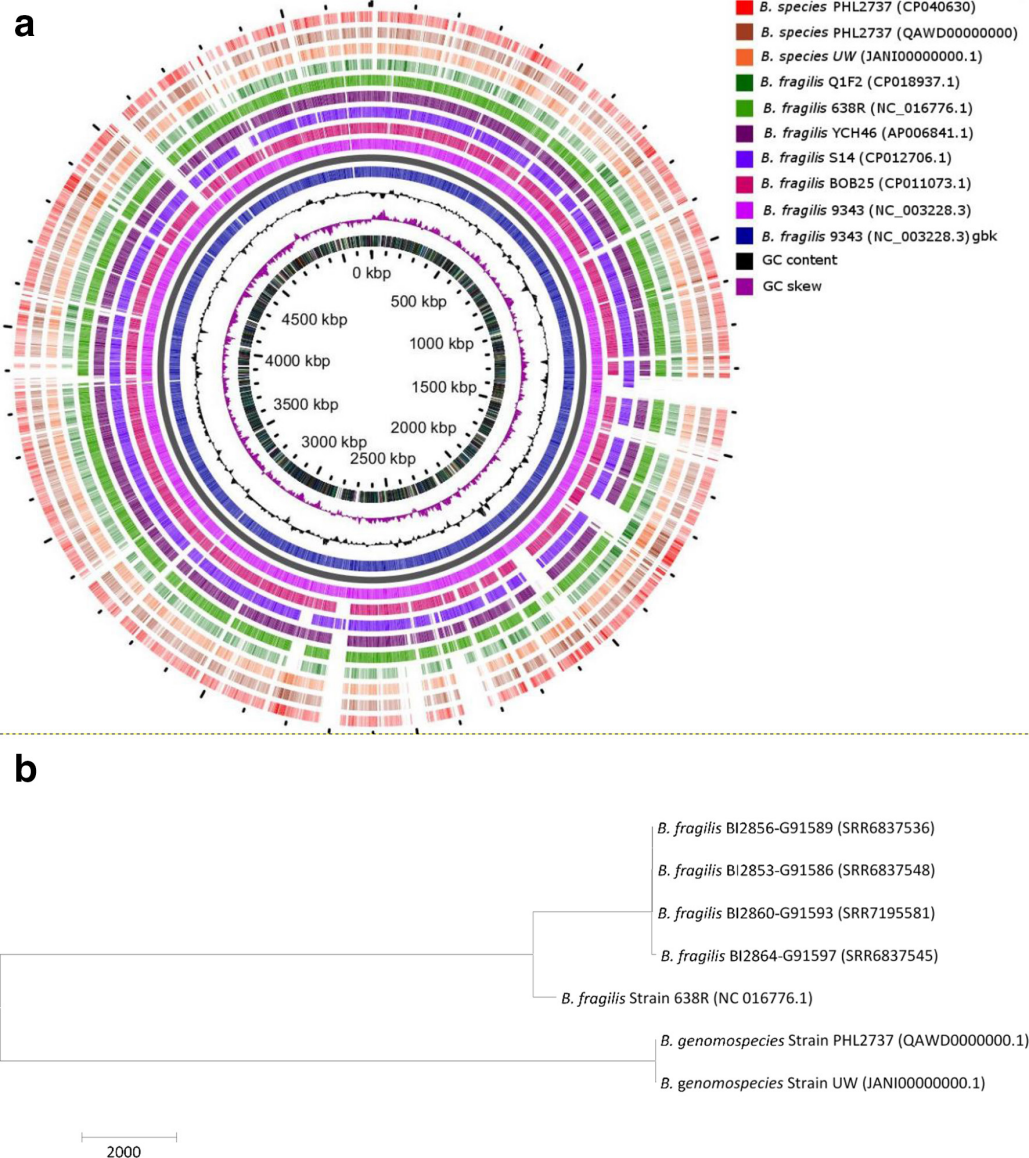
Genome atlas diagrams of *Bacteroides* spp. PHL2737 (QAWD00000000) from this study compare to other *B. fragilis *. *B. fragilis* strain NCTC 9343 was used as a reference sequence. A lack of colour represents the absence of genes in the genome of the corresponding strain at that position. Phylogenetic analysis of single nucleotide variation (SNV) of PHL2737. Core-genome SNV-based phylogenetic tree of *Bacteroides* genomospecies PHL2737 (accession no. QAWD00000000.1) and closely related GenBank isolates. SNV were called using smalt 0.7.4. *B. fragilis* strain 638R was used as a reference. The meta-alignment of informative core SNV positions was used to create a maximum likelihood phylogenetic tree using mega 6 on 28 233 core high-quality SNVs. Maximum likelihood phylogeny indicates the presence of two clusters separated by >25 000 SNVs. One cluster contains four GenBank isolates, with 8–107 SNV differences within each other and >3000 SNVs to reference. The second cluster contains GenBank isolate JANI01000001.1 and clinical isolate PHL2737 with only 8 SNV differences.

An average nucleotide identity (ANI) value <90 % was obtained between PHL2737 and the *B. fragilis* reference sequences (Table 1). It is recommended that an ANI cut-off value >96 % is required for definitive species-specific identification [3]. Therefore, using ANI values, species determination was performed by comparison of the full genome of PHL2737 and reference genomes in NCBI. Results demonstrated that PHL2737 was most similar to the previously published *Bacteroides* genomospecies UW isolate 1 (strain JANI00000000.1) with ANI values of 99.97 % (Table 1). The genome atlas plot shows high similarities between PHL2737 and UW isolate 1 compared reference *B. fragilis* strains (Fig. 1).

**Table 1.** Average nucleotide identify (ANI) of PHL2737 against other *Bacteroides* spp.

* Bacteroides* strains	1	2	3	4	5	6	7	8	9
1.PHL2737 (QAWD00000000, CP040630)	100								
*2. Bacteroides* ***spc* UW (JANI00000000.1**)	99.97	100							
*3. B. fragilis* **Q1F2 (CP018937.1**)	98.25	98.16	100						
*4. B. fragilis* **NCTC 9343 (NC_003228.3**)	87.21	87.17	87.16	100					
*5. B. fragilis* **638R (NC_016776.1**)	87.06	87.24	87.05	99.09	100				
*6. B. fragilis* **BOB25 (CP011073.1**)	87.25	87.17	87.38	99.08	99.12	100			
*7. B. fragilis* **YCH46 (AP006841.1**)	87.08	87.27	87.27	99.08	99.02	99.08	100		
*8. B. fragilis* **S14 (CP012706.1**)	86.81	86.88	87.33	99.37	99.11	99.09	99.21	100	
*9. B. fragilis* **BFBE1 (LN877293.1**)	87.17	8725	87.36	99.11	99.16	99.17	99.17	99.15	100

We also examined full-length 16 s rRNA and *cpn60* gene sequences from the *de novo* assembly to verify the identification. blast results showed a 99 % identity of PHL2737 16S rRNA gene to *B. fragilis* strains Q1F2, BOB25, YCH46 and 638R. The *cpn60* gene sequences were 99 % identical only to *B. fragilis* Q1F2, while only 89 % identical to BOB25, YCH46 and 638R.

The full genome of *B. fragilis* 638R (NCBI accession no. FQ312004.1) was chosen as the reference strain to compare PHL2737. High-quality core SNVs were called using a custom pipeline [4, 5]. The meta-alignment of informative core SNV positions was used to create a maximum likelihood (ML) phylogenetic tree using mega 6 [5] (Fig. 1b). The ML phylogeny using core SNVs grouped the analysed sequences into two clusters; a branch containing reference 638R was separated by >25 000 SNVs from PHL2737. There were only eight SNVs observed between PHL2737 and strain UW (JANI00000000.1), further demonstrating a close genetic relatedness of the two strains.

Together, these results suggest that PHL2737 is not *B. fragilis sensu strictu* as suggested by MALDI-ToF MS and 16S rRNA gene sequencing, but rather a member of a novel species within the *B. fragilis* group to which the *Bacteroides* genomospecies UW strain also belongs. Interestingly, our analysis also shows that the Q1F2 strain is most similar to PHL2737 and UW strain, and thus should also be part of this novel genomospecies. This data demonstrates the utility of WGS for accurate identification and characterization of challenging bacterial species. Of note, the *Bacteroides* UW strain, also MDR, was recovered from a patient who had travel and had hospitalization history in India. Our patient had not left Canada in the previous 5 years though had emigrated from India many years prior to presentation.

To identify antimicrobial-resistant genes, the WGS of PHL2737 was interrogated using ResFinder 2.1 [6]. Several known genes involved in antimicrobial resistance were identified: *nimE* (metronidazole)*, tetQ* (tetracycline)*, cfiA13* (beta-lactams) and *erm(F*) (clindamycin). Additionally, a mutation in *gyrA* causing a S82F substitution causing fluoroquinolone resistance was also identified.

**Fig. 2.**
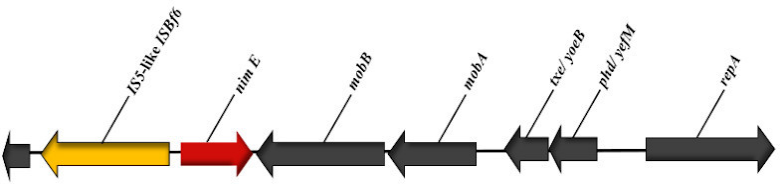
Linear schematic representation of region containing the *nimE* gene and surrounding ORFs in plasmid 1.

The Nanopore sequencing revealed PHL2737 had three plasmids (8331 bp, 5595 bp and 2750 bp) none of which had corresponding complete entries in the NCBI database. It is not clear, based on publically available sequences, whether the UW strain contained plasmids or not as the *nimE* gene was not found on the chromosomal sequences of the UW strain. The *nimE* gene on the PHL2737 strain was found on one of the plasmids (Fig. 2).

Both *nimE* or *cfiA* genes have been reported in several phenotypically metronidazole-sensitive strains of *Bacteroides* spp. implying that their presence may not necessarily confer resistance [7]. However, the presence of a specific insertion sequence (IS) element upstream of the promoter responsible for transcribing these genes appears to be necessary for up-regulation and expression of these proteins resulting in the metronidazole-resistant phenotype [8]. Using rast, we were able to locate *nimE* and the associated upstream insertion sequence *IS5*-like element (*ISBf6* family transposase) on the largest plasmid (plasmid 1) (Fig. 2). The presence of the IS upstream of *nimE* coupled with phenotypic resistance to metronidazole supports the role of *NimE* in metronidazole resistance. Similarly, the metallo‐β‐ lactamase gene, *cfiA13* and its *IS1380*-like element (IS613 family transposase) were found on the bacterial chromosome, suggesting this as the mechanism of β‐lactam/carbapenem resistance [7].

Since 2005, reports of MDR *B. fragilis* from Britain, Kuwait, Greece, Hungary, Denmark and three from the USA have been reported [9–13]. Antimicrobial resistance should be considered for serious *Bacteroides* infections that are not responding to typical therapies such as metronidazole, piperacillin-tazobactam and carbapenems in conjunction with source control. MDR *Bacteroides* spp. with resistance to metronidazole and carbapenems are present in Canada and should be a consideration when treatment failures are observed with empiric therapy for *Bacteroides* spp. infections.

### Conclusions

We identified the first MDR *Bacteroides* spp. causing invasive infection in Canada. Characterization of this strain using WGS data revealed that this strain is a novel *Bacteroides* genomospecies carrying a plasmid harbouring the *nimE* gene, linked to metronidazole resistance. The presence of the *nimE* gene on a mobile genetic element is of great concern due to the possibility of horizontal gene transfer [14].

With an increasing number of reports of MDR *B. fragilis* strains within the past decade, our findings further highlight the importance of ongoing surveillance to guide empiric antimicrobial therapy and to track prevalence of resistance among anaerobic bacteria.
